# Differences in Pressure Pain Threshold and Strain Elastography Between Women with and Without Fibromyalgia: A Cross-Sectional Study

**DOI:** 10.3390/diagnostics16040559

**Published:** 2026-02-13

**Authors:** María Aguilar-García, María Encarnación Aguilar-Ferrándiz, Ana González-Muñoz, Santiago Navarro-Ledesma

**Affiliations:** 1 Biomedicine PhD Program, Faculty of Health Sciences, University of Granada, 18060 Granada, Spain; 2 Department of Physical Therapy, Instituto de Investigación Biosanitaria Granada (IBIS.Granada), University of Granada, 18060 Granada, Spain; 3 Department of Physiotherapy and Sport Sciences, Faculty of Biomedical and Sport Sciences, Universidad Europea de Andalucía, 29010 Malaga, Spain; 4 Clínica Actium, Avenida Hernán Núñez de Toledo 6, 29018 Malaga, Spain; 5 Department of Physical Therapy, Faculty of Health Sciences, Research Unit of Excellence of the Melilla University Campus (UECUMEL), Melilla Campus, University of Granada, 52004 Melilla, Spain

**Keywords:** fibromyalgia, elasticity imaging techniques, pain threshold, soft tissue

## Abstract

**Background**: Fibromyalgia (FM) is a chronic pain condition primarily linked to central sensitization, although peripheral tissue-related factors have also been suggested. Ultrasound strain elastography (SEL) provides a semi-quantitative, operator-dependent estimate of tissue deformation under standardized compression, yet evidence comparing SEL findings and pressure pain sensitivity between FM and healthy controls at standardized tender-point sites remains limited. **Objective**: To compare pressure pain threshold (PPT) and SEL-derived tissue deformation between women with FM and healthy controls across standardized FM tender-point sites. **Methods**: In this cross-sectional study, 84 women (42 with FM; 42 healthy controls) were recruited from a private rehabilitation center in Málaga (Spain). PPT and SEL were assessed bilaterally at 13 standardized tender-point sites. Between-group differences were examined using Student’s *t*-test or the Mann–Whitney U test according to distribution. **Results**: Women with FM exhibited lower PPT across all assessed sites (*p* < 0.01) and lower SEL-derived deformation scores at most sites, whereas no between-group SEL differences were observed at the dominant and non-dominant forearm, non-dominant lower cervical region, dominant paraspinal region, and bilateral lateral pectoral region. **Conclusions**: Compared with controls, women with FM showed reduced pressure pain thresholds and site-dependent differences in SEL-derived tissue deformation at standardized tender-point sites. Given the cross-sectional and exploratory design, SEL findings should be interpreted cautiously and considered non-diagnostic; heterogeneity across anatomical sites should be considered in future confirmatory and longitudinal studies.

## 1. Introduction

Fibromyalgia (FM) is a syndrome of uncertain etiology characterized by chronic widespread musculoskeletal pain, accompanied by symptoms such as dysesthesia, fatigue, morning stiffness, tender points (TPs), headache, sleep disturbances, anxiety, and cognitive impairments. Although FM is primarily conceptualized as a central sensitization syndrome, peripheral tissue-related factors have been proposed as potential contributors or secondary adaptations to persistent pain, which remains an unresolved issue [1,2,3,4,5].

The prevalence of fibromyalgia varies depending on the diagnostic criteria applied [6]. The most commonly used diagnostic framework is established by the American College of Rheumatology (ACR), which has evolved over time from tender point–based approaches to symptom-based criteria, including widespread pain lasting for at least three months, pain elicited by 4 kg of digital pressure, and widespread tenderness assessed through a standardized map of characteristic musculoskeletal tender points [4,6,7,8]. According to the latest 2016 update by ACR, the diagnostic criteria for FM must include widespread pain in at least 4 of 5 body regions, symptoms or pain persisting for at least 3 months, or elevated scores on the Widespread Pain Index (WPI) or the Symptom Severity Scale (SSS), indicating a worsening in both the extent and intensity of pain [3]. Although these criteria support the differential diagnosis of FM from other rheumatic and pain-related disorders, they do not exclude the possibility of coexisting conditions [3]

The global prevalence of FM ranges from 2 to 3% [6,9], with reported rates in Europe varying between 2.1 and 2.4%. In Spain, the prevalence is 4.2% among women and 0.2% among men [9,10,11,12,13,14], with the highest frequency observed in individuals aged 50 to 59 years [11,12,15,16,17].

Although the pathophysiological origin of FM remains unclear, current evidence supports its classification as a central sensitization syndrome [6]. Central sensitization is defined as a mechanism of amplified neuronal signaling within the central nervous system (CNS) [6,18], resulting in heightened pain perception. This phenomenon involves, among other factors, dysfunction of the hypothalamic–pituitary–adrenal (HPA) axis, which plays a key role in modulating stress responses and regulating neuroendocrine processes associated with pain [6,18,19,20,21,22]. Both ascending and descending pain pathways, as well as neurotransmitter systems, may be altered, which contributes to the presence of hyperalgesia and allodynia in individuals with fibromyalgia [6,18,20,21]. In parallel, peripheral sensitization refers to an augmented response of peripheral nociceptors, potentially increasing nociceptive input and contributing to site-specific pain sensitivity [6,16,20,21,23,24,25].

From a conceptual standpoint, central sensitization is considered a primary driver of widespread pain in FM, whereas peripheral tissue alterations—if present—may act as modulators, local amplifiers, or secondary adaptations to chronic pain and altered autonomic/inflammatory milieu. Moreover, this condition of central sensitization and chronic pain involves a low-grade inflammatory state and alterations in brain regions responsible for pain processing and behavioral regulation [5,26,27]. On the other hand, peripheral sensitization involves an augmented response of peripheral nociceptors, which leads the neuronal system to progressively respond to increasingly milder stimuli [6]. Previous studies have investigated and demonstrated the relationship between psychological factors and autonomic symptoms in individuals with FM [5,6,26,28,29]. Low-grade inflammation present in patients with FM, along with sympathetic nervous system activity, affects the elasticity of soft tissue and even cartilaginous tissue [5,30,31,32,33,34].

Numerous techniques, including magnetic resonance imaging (MRI), microanalytical techniques, infrared thermography, and ultrasonography, have been employed to quantify and assess myofascial pain in FM [6,35]. Recently, ultrasound-based techniques such as strain elastography have emerged, facilitating a more accessible and clinically applicable assessment of tissue-related pain features [36,37,38]. However, ultrasound elastography encompasses different modalities with distinct outputs; therefore, specifying the technique is essential. In particular, strain elastography (SEL) provides an operator-dependent, semi-quantitative estimate of tissue deformation under controlled compression, which differs conceptually and metrically from shear-wave elastography [36,39]. A recent study analyzed the correlations between psychological factors and SEL-derived tissue deformation patterns in patients with FM [40].

Previous studies have shown a relationship between the presence of tender points (TPs) and musculoskeletal disorders such as FM. A tender point (TP) can be defined as a specific localized area that may be found in muscle tissue, the muscle–tendon junction, fat pads, or the bursal region, and is characterized by extreme sensitivity to touch or pressure [23,35,41]. The term “TP” is used to describe areas where the application of local pressure elicits pain without radiation or reproduction of referred pain. TPs are commonly located in the back, face, neck, knees, calves, gluteus maximus, quadriceps, and hips [35,42]. In line with this, correlations between the pressure pain threshold (PPT) at characteristic FM pain points and tissue deformation pattern assessed by SEL have been studied in patients with FM [37].

Despite increasing interest in SEL and peripheral tissue characterization in FM, prior elastography literature remains heterogeneous regarding anatomical targets and elastography modalities, and evidence directly comparing SEL measures between women with FM and healthy controls at standardized FM tender-point sites, while concurrently quantifying pain sensitivity using PPT, remains scarce. Although previous studies have explored SEL-based tissue deformation responses at characteristic pain points in individuals with FM as potential outcome measures in relation to pain and psychological factors [37,40], there are currently no studies that have compared SEL-based measures with PPT between healthy individuals and patients with FM across standardized tender-point sites.

Therefore, the objective of the present study is to compare the PPT and SEL-derived deformation measures between a FM group and a healthy control group at the characteristic pain points described in patients with FM. It was hypothesized that women with FM would present lower PPT values and differences in SEL measures compared with healthy controls, reflecting differences in tissue deformation under compression rather than direct evidence of tissue pathology.

## 2. Materials and Methods

### 2.1. Study Design

This was a cross-sectional observational study. Ethical permission was approved by the Ethics Committee of Human Research at the University of Granada, Spain (approval number: 1044/CEIH/2020). The study was conducted in accordance with the principles of the Declaration of Helsinki and in line with the Statement of Recommendations for Observational Studies (STROBE).

### 2.2. Setting

The total study sample, consisting of 84 participants, was recruited from a private rehabilitation center in the province of Málaga, Spain, using a convenience sampling approach based on voluntary participation.

### 2.3. Sample

A sample of 84 women participated in this study. Forty-two women comprised the control group, and forty-two were assigned to the fibromyalgia group. Participants in the FM group did not receive any type of treatment in order to avoid potential interference with the measurements collected.

A physiotherapist was responsible for recruiting participants at a private center in Málaga and for assigning them to their respective groups. Compliance with the inclusion and exclusion criteria for group allocation was also supervised by a physiotherapist. Participation in the study was voluntary, and only women were included to reduce sex-related variability in pain sensitivity and SEL-derived deformation measures.

All SEL and PPT measurements were performed by a single experienced evaluator. However, intra-observer reliability was not formally assessed in the present study.

The evaluator was not blinded to group allocation. Limb dominance was determined by self-report, asking participants to indicate the upper limb they preferentially used for daily activities.

#### 2.3.1. Healthy Group

Inclusion criteria

- Aged between 18 and 64 years.
- Participants not diagnosed with fibromyalgia.

Exclusion criteria

- Presenting any inflammatory, orthopedic, or neurological disorder.

#### 2.3.2. Fibromyalgia Group

Inclusion criteria

- Aged between 18 and 64 years.
- Being diagnosed with fibromyalgia according to the ACR classification criteria.

Exclusion criteria

- Presenting any inflammatory, orthopedic, or neurological disorder.

### 2.4. Outcome Measures

#### 2.4.1. SEL-Derived Tissue Deformation Assessment

**Figure 1:**
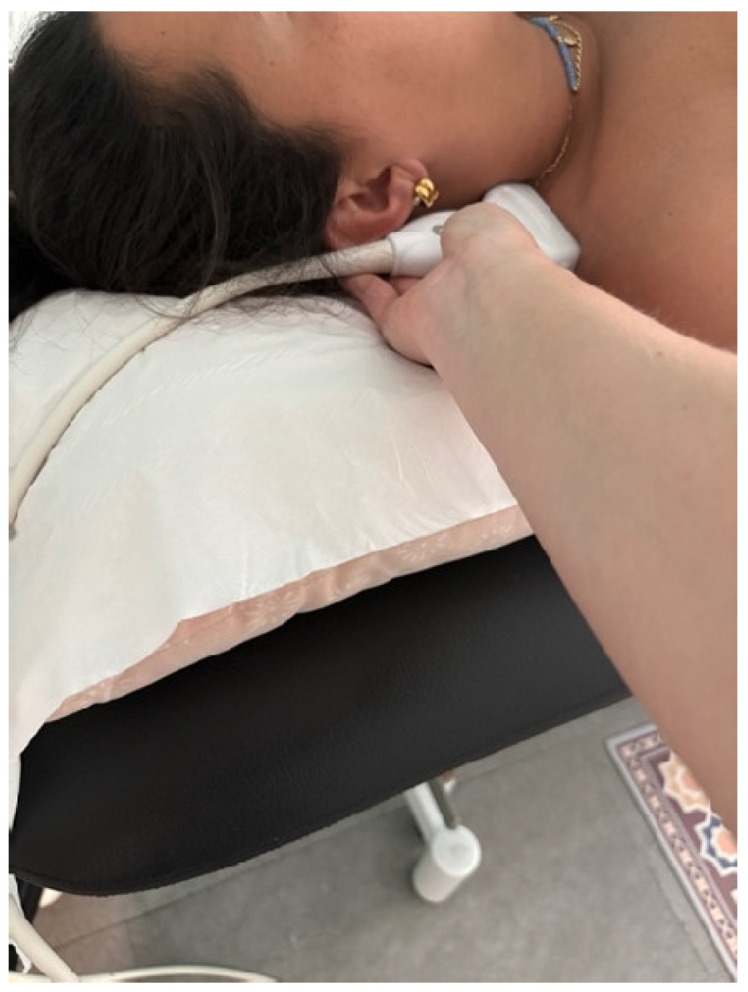
Strain elastography performed at the central portion of the upper trapezius muscle.

Ultrasound strain elastography (SEL) was used to assess relative soft-tissue deformation behavior under compression at standardized tender-point sites. All measurements were performed by a physiotherapist with 11 years of experience in ultrasound imaging, using a Logiq S7 device with a 15 MHz linear probe (GE Healthcare, Milwaukee, WI, USA), acknowledging the operator-dependent nature of the technique. The transducer was placed longitudinally along the muscle fibers, with the center of the probe positioned over the tender points (TPs) in the regions previously described [43] and with the patient positioned according to the established TP identification protocol (Figure 1) [44,45].

Compression magnitude was monitored and standardized using the system’s built-in quality control (“dedicated software”), which displays compression adequacy via green bars; only sequences meeting the recommended quality (green bars) were retained for analysis. The recommended compression size was approximately 2–5 mm. The elastography color scale displayed red (hard), green (medium), and blue (soft) tissue patterns, accompanied by a reference bar with ‘S’ (soft) at the top and ‘H’ (hard) at the bottom.

**Figure 2:**
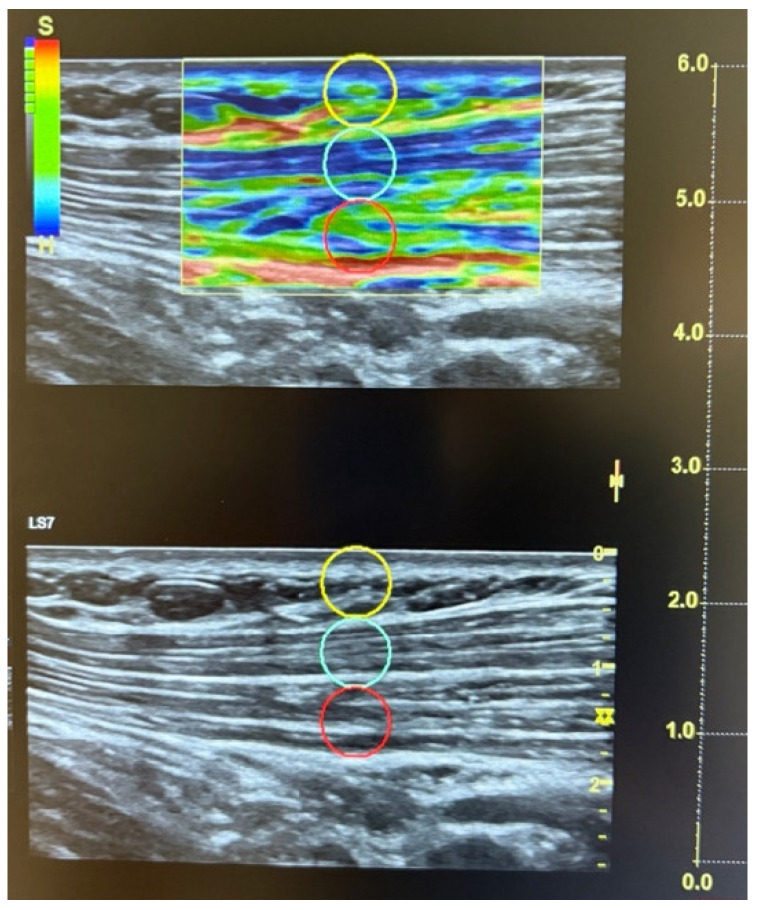
Quantitative strain elastography (SEL) was used to assess the upper trapezius muscle in a woman with fibromyalgia. The various circles represent distinct regions of interest (ROIs), corresponding to different tissue depths from superficial to deep layers.

SEL values ranged from 0 to 6 (0 = greater deformation; 6 = lower deformation) according to the manufacturer’s algorithm, reflecting semi-quantitative deformation estimates rather than direct measures of tissue stiffness or pathology. The exact raw strain value was calculated using a standardized 5 mm circular region of interest (ROI) placed within the selected area, following manufacturer instructions and previous studies (see Figure 2). Three high-quality acquisitions were obtained per site, and the mean of the three measurements was used to minimize intra-observer variation. Following the ACR criteria, a total of twenty-six points—thirteen on the dominant side and thirteen on the non-dominant side—were assessed [15]: (i) suboccipital muscle insertions; (ii) anterior aspect of the lower cervical intertransverse space (C5–C7); (iii) midpoint of the trapezius muscle (upper border); (iv) supraspinatus origin, above the scapular spine near the medial border; (v) paraspinal region at mid-scapular level, 3 cm lateral to the midline; (vi) second costochondral junction (second rib); (vii) level of the fourth rib at the anterior axillary line (lateral pectoral region); (viii) 2 cm distal to the epicondyle; (ix) medial epicondyle; (x) distal dorsal third of the forearm; (xi) upper outer quadrants of the buttocks, in the anterior fold of the gluteal muscle; (xii) greater trochanter, just posterior to the trochanteric prominence; and (xiii) medial fat pad of the knee.

#### 2.4.2. Pain Pressure Threshold

A standard pressure algometer with a contact surface of 1 cm^2^ (FPK 20; Wagner Instruments, Greenwich, CT, USA) was used to measure the pressure pain threshold (PPT) at the 13 points on each side identified as characteristic of fibromyalgia according to the ACR consensus [15], following the established protocol [46].

PPTs were measured at the exact same points where tissue deformation was assessed using SEL. Standardized instructions were provided prior to testing to reduce potential learning effects, and all measurements were conducted by a physiotherapist with prior specific training. At each site, three consecutive PPT measurements were obtained and the mean value was used for analysis to improve measurement stability and reduce random error. The analyses performed using ultrasound strain elastography and pressure pain threshold were purely exploratory in nature.

### 2.5. Statistical Analysis

SPSS Statistics software version 24 for Windows (IBM Corporation, Armonk, NY, USA) was used for statistical analysis. The Shapiro–Wilk test was applied to assess the normality and distribution of the variables. Descriptive variables with a Shapiro–Wilk *p*-value > 0.05 were expressed as mean ± standard deviation (SD), while those with a *p*-value < 0.05 were presented as median and interquartile range (IQR). Descriptive variables are shown in Table 1. Descriptive data for PPT and SEL are presented in Table 2 and Table 3, respectively. To analyze between-group differences, Student’s *t*-test was applied to variables with Shapiro–Wilk *p* > 0.05, and the Mann–Whitney U test was used for variables with Shapiro–Wilk *p* < 0.05. Group differences in PPT are presented in Table 4. Group differences in SEL are shown in Table 5.

Because multiple anatomical sites were analyzed, an increased Type I error (false-positive) risk due to multiple comparisons was considered. Therefore, *p*-values were adjusted using false discovery rate (FDR) control (Benjamini–Hochberg; q = 0.05) across site-level tests. For *p*-values reported as <0.001, a conservative value of 0.001 was used for the adjustment. Effect sizes (Cohen’s d) were additionally reported to quantify the magnitude of between-group differences independently of statistical significance. All analyses were conducted on a site-specific basis. No averaging of dominant and non-dominant sides was performed at any stage of the analysis. These effect sizes quantify between-group differences and do not imply causality in this cross-sectional design. PPT was treated as the primary outcome of the study, and all analyses involving SEL were predefined as exploratory and site-specific.

### 2.6. Sample Size

The sample size was estimated using G*Power version 3.1.9.7 (Heinrich Heine University, Düsseldorf, Germany). Based on data from a previous cross-sectional study that assessed pressure pain threshold via sonoelastography in individuals with nonspecific lumbopelvic pain<sup>31</sup>, a minimum of 18 participants with FM was required to achieve a statistical power of 80% with an alpha level (α) of 0.05 [47]. Given the exploratory nature of the present study and the intention to perform between-group comparisons across multiple anatomical sites, the target sample size was increased to enhance statistical precision and robustness. Ultimately, the final sample included a total of 84 women, with 42 assigned to the control group and 42 diagnosed with FM.

## 3. Results

### 3.1. Sociodemographic Characteristics

**Figure 3:**
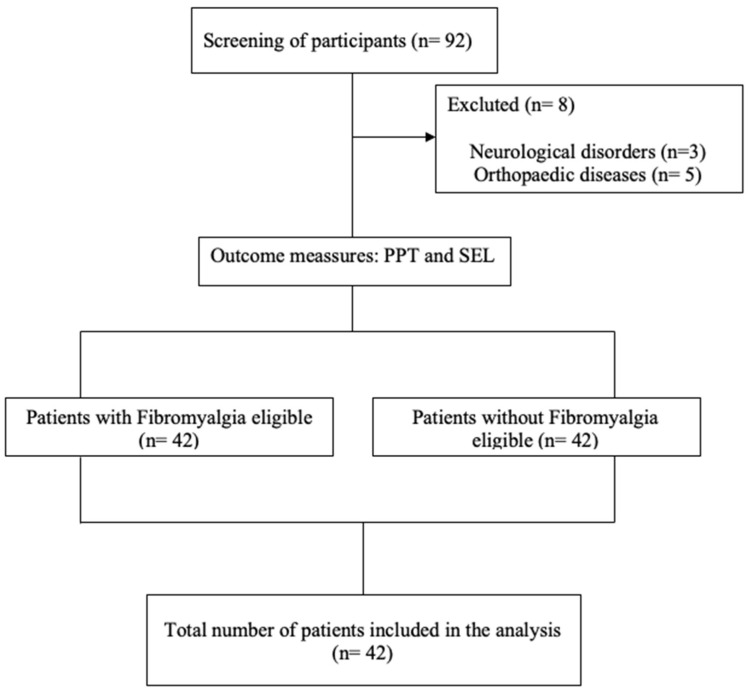
Flow diagram of participants. Abbreviations: PPT (pressure pain threshold); SEL (Ultrasound strain elastography).

The sociodemographic characteristics are shown in Table 1, Table 2 and Table 3. Eighty-four women participated in the study. A total of 42 participants were included in the control group and 42 in the FM group. All participants met the inclusion and exclusion criteria established for this study. A flow diagram has been included (Figure 3). Effect sizes (Cohen’s d) are reported in Table 4 and Table 5 to quantify the magnitude of between-group differences across anatomical sites.

### 3.2. Differences in PPT and SEL Between the Control Group and the Fibromyalgia Group

Table 4 presents the between-group differences in PPT variables analyzed using Student’s *t*-test and the Mann–Whitney U test. To address the increased Type I error (false-positive) risk associated with multiple site-level comparisons, *p*-values were additionally adjusted using false discovery rate control (Benjamini–Hochberg; q = 0.05). Results for PPT and SEL are presented in Table 4 and Table 5, respectively. All PPT comparisons remained significant after FDR correction.

Table 5 presents Student’s *t*-test results and Mann–Whitney U test results for between-group differences in SEL variables. To address the increased Type I error (false-positive) risk associated with multiple site-level comparisons, *p*-values were additionally adjusted using false discovery rate control (Benjamini–Hochberg; q = 0.05). Results for PPT and SEL are presented in Table 4 and Table 5, respectively.

## 4. Discussion

The present study aimed to investigate whether differences in pressure pain thresholds at TPs and in SEL measures exist between women with and without FM. All findings should be interpreted cautiously given the cross-sectional, exploratory design, the heterogeneous anatomical targets assessed, and the increased risk of Type I error due to multiple testing across sites. Women in the FM group exhibited markedly lower PPT values than the control group across all assessed tender-point sites. Beyond statistical significance (*p* < 0.01 at all sites), the magnitude of these differences was consistently very large (Cohen’s d range −2.18 to −3.78), supporting robust pressure pain hypersensitivity across regions.

With respect to SEL, between-group differences were site-dependent, and the pattern was notably less uniform than that observed for PPT. It is important to note that SEL reflects operator-dependent, imaging-based tissue deformation responses under compression rather than direct measures of tissue stiffness or elasticity. To mitigate Type I error, *p*-values were additionally adjusted using false discovery rate control (Benjamini–Hochberg). After FDR correction, SEL between-group differences remained significant at most sites (19/26), with effect sizes ranging from negligible to large across locations (Cohen’s d −0.07 to −1.93). No significant SEL differences were observed at the forearm (dominant and non-dominant), lower cervical region (non-dominant), paraspinal region (dominant and non-dominant), and lateral pectoral regions (dominant and non-dominant).

### 4.1. Comparison with Other Studies

The data presented in this study support the presence of marked alterations in pressure pain sensitivity in fibromyalgia and suggest site-dependent differences in ultrasound strain elastography (SEL) measures. The statistically significant results found when analyzing between-group differences in pressure pain thresholds were consistent with previous literature, which described FM as a condition characterized by enhanced pain perception, reduced pain thresholds, hyperalgesia, and allodynia [6,18,21]. Previous studies used the same protocol to measure pressure pain thresholds in women with FM [14,22,37]; however, to the best of current knowledge, the concurrent application of this PPT protocol together with SEL in both the FM group and a healthy control group across a standardized tender-point panel has been scarcely investigated. Although the pathophysiological processes responsible for fibromyalgia are still being investigated, the condition is currently defined as a central sensitization syndrome based on the available evidence [6,18,24]. Alterations in functional neuroimaging findings and in neurotransmitters involved in sensory and pain transmission have been reported in patients with fibromyalgia, indicating the presence of widespread hyperalgesia and/or allodynia [21,24,48]. These central alterations may help explain the reduced tolerance to pressure pain thresholds observed in the FM group.

Regarding SEL findings, it should be emphasized that this technique provides an operator-dependent, imaging-based representation of tissue deformation under compression rather than a direct measurement of intrinsic tissue stiffness, elasticity, inflammation, or pathology. Accordingly, SEL findings cannot be used to infer specific neurochemical or pathophysiological mechanisms. Previous studies have reported alterations in neurotransmitter systems in FM, including increased levels of excitatory mediators and reduced inhibitory modulation [49,50,51,52]; however, these mechanisms are related to central pain processing and should be interpreted independently of SEL-derived measures.

Due to alterations in the central nervous system, in addition to central sensitization, patients with fibromyalgia are also characterized by neurovegetative symptoms and peripheral sensitization, as well as comorbidities such as irritable bowel syndrome, gynecological disorders, chronic fatigue syndrome, postural orthostatic tachycardia, circadian variations in blood pressure, and an exaggerated response to auditory, thermal, or mental stimuli [5,21,22,25,34,53,54,55]. Inflammatory processes are also known to be present in both peripheral structures and within the central nervous system in relation to fibromyalgia [6,56,57].

Within this complex clinical context, alterations in the central nervous system, along with autonomic symptoms and the low-grade inflammation observed in fibromyalgia, may indirectly influence peripheral tissue behavior. However, it should be emphasized that provides a semiquantitative assessment of tissue deformation under compression and does not allow direct inferences regarding inflammatory status, autonomic dysfunction, or peripheral pathology. Therefore, any observed between-group differences in SEL-derived measures should be interpreted as secondary and non-specific adaptations, rather than as evidence of primary peripheral pathology [5,30,31,32,34].

In the present data, SEL findings were heterogeneous across anatomical sites: lower SEL scores were observed at most sites, whereas several regions showed no statistically significant differences. Importantly, effect-size estimates for SEL ranged from negligible to large (Cohen’s d approximately −0.07 to −1.93), underscoring site-dependent variability rather than a uniform peripheral pattern. The points that did not show significant differences between groups were the dominant and non-dominant forearm, non-dominant lower cervical region, dominant paraspinal area, and both dominant and non-dominant lateral pectoral regions. This heterogeneity warrants further investigation. A possible hypothesis is that regional loading and habitual use (particularly in upper-limb sites) may contribute to more control-like SEL patterns in some locations; however, this interpretation remains speculative and should be tested by incorporating objective physical activity and muscle-function measures [5,37,43]. In this line, future research should consider evaluating the level of physical activity in both groups and exploring potential correlations between physical activity, SEL measures, and pressure pain thresholds. This approach may help clarify the influence of functional movement on peripheral tissue characteristics and pain sensitivity in individuals with FM.

Comparison with previous studies remains limited, as evidence directly comparing SEL measures between women with and without FM is methodologically heterogeneous across elastography modalities and anatomical targets. However, between-group elastography comparisons have been explored using different techniques, but significant differences between groups were not found. These authors employed techniques different from those used in the present investigation. In their studies, Muro-Culebras and Cuesta-Vargas used sonomyography and sono-myelastography to measure stiffness at trigger points, whereas Karayol and Sibel applied shear wave elastography to assess elasticity in the rhomboid major muscle [35,58]. Such methodological diversity (e.g., strain vs. shear-wave approaches and differing anatomical targets) limits direct comparability and may partially account for discrepant findings.

It is important to highlight that the use of strain elastography to non-invasively assess tissue deformation behavior has gained increasing attention in recent years. Numerous studies have demonstrated its usefulness for the analysis of various anatomical structures, as well as a tool for evaluating the effectiveness of different interventions [14,36,38,59,60,61,62]. Therefore, the findings presented here, although contrary to those reported by Muro-Culebras and Cuesta-Vargas as well as by Karayol and Sibel, should be interpreted cautiously in light of differences in elastography modality and the exploratory nature of multi-site testing. Lower SEL scores in the FM group suggest differences in strain elastography outputs under standardized compression; however, SEL provides a semi-quantitative deformation-based measure and should not be overinterpreted as a direct indicator of “tissue stiffness” in the biomechanical sense. The present findings suggest that differences observed in SEL-derived measures coexist with well-established alterations in central pain processing in FM, without implying a uniform or specific peripheral tissue mechanical signature. Accordingly, the present results do not support the use of SEL as a mechanistic or diagnostic biomarker in FM.

Although alterations in the central nervous system may induce changes in the tensegrity of peripheral tissue structures, the systems involved in pain processing appear to be primarily responsible for the increased pain response observed in individuals with fibromyalgia. While this idea is consistent with current evidence, which links the pathophysiology of fibromyalgia to central sensitization, it should be interpreted cautiously given the operator-dependence, semi-quantitative nature of strain elastography and the absence of intra-/inter-rater reliability assessment in the present study. Strain elastography assessments are operator-dependent; further studies are required to confirm our findings. Also, it would be important to investigate whether other factors could explain the lower SEL values observed in the FM group compared to the control group, such as differences in physical activity levels, muscular condition, or related physiological variables, and whether these factors are correlated with pressure pain thresholds.

### 4.2. Clinical Application

These cross-sectional findings do not support diagnostic, prognostic, or monitoring use of strain elastography (SEL) in fibromyalgia. Nevertheless, SEL may be considered a complementary research tool to characterize tissue deformation response alongside clinical assessment and pain sensitivity measures but not as a tool for diagnosis, prognosis, or mechanistic inference.

The observed between-group differences in PPT and the site-dependent SEL pattern suggest that tissue deformation behavior under standardized compression may differ between the FM group and the control group at selected anatomical sites. However, SEL provides a semi-quantitative, deformation-based output and should not be overinterpreted as a direct measure of tissue stiffness or “elastic properties” in a biomechanical sense.

Although central sensitization remains the primary mechanistic framework for FM, the present SEL findings are compatible with the presence of differences in tissue deformation responses under compression and/or secondary adaptations to chronic pain; however, no causal or pathophysiological inferences can be drawn from this cross-sectional design.

The potential role of SEL in FM should therefore be framed as exploratory, and any clinical application (e.g., phenotyping or subgrouping) requires confirmation in adequately powered studies that include standardized acquisition protocols and formal intra- and inter-rater reliability assessment.

Longitudinal and interventional designs are also required before considering SEL for monitoring disease course or treatment response. Future research should determine whether SEL patterns are reproducible across operators and devices, and whether they relate to clinical outcomes when potential confounders (e.g., physical activity, muscle condition, and clinical management) are measured and controlled. Only then can the utility of SEL as a biomarker candidate for progression or therapeutic response be appropriately evaluated.

### 4.3. Strengths

It is important to emphasize several strengths of this study. To our knowledge, it is the first investigation to systematically evaluate SEL-derived tissue deformation measures across 26 standardized anatomical sites in women with FM compared with healthy controls. The combination of SEL with PPT measurements allowed for a more comprehensive characterization of the peripheral and sensory alterations associated with FM. All assessments were conducted by a physiotherapist with extensive experience in musculoskeletal ultrasound, ensuring methodological consistency, and triplicate measurements were taken at each site to enhance data reliability.

### 4.4. Limitations

Several limitations should be acknowledged. First, the cross-sectional design precluded the establishment of causal relationships between SEL-derived tissue deformation measures, PPT and central sensitization. Accordingly, no temporal, mechanistic, or casual inferences can be draw from the present findings.

Assessor were not blinded to group allocation, which may have introduced measurement bias. Although all assessments were performed by an experienced physiotherapist, the absence of assessor blinding represents a methodological limitation.

Although the sample size was sufficient to detect statistically significant differences, its magnitude may limit the generalizability of the findings to other populations.

In addition, SEL is an operator-dependent imaging technique and remains sensitive to variations in transducer placement and applied compression, even when standardized protocols are used.

Intra- and inter-rater reliability for SEL acquisition and interpretation was not assessed, which limits reproducibility and may contribute to variability across anatomical sites.

Furthermore, although FDR control was applied, the analysis involved a large number of site-level comparisons and the results should therefore be considered multiple anatomical sites and should be considered hypothesis-generating. Residual Type I error risk and site-dependent heterogeneity cannot be fully excluded. Accordingly, all site-specific SEL analysis should be interpreted as exploratory and hypothesis-generating, whereas PPT findings represent the primary outcome of the present study.

Only women were included in the present study, which limits the applicability of the findings to male patients with FM.

Finally, the absence of systematically collected data on physical activity levels, occupational exposure, pharmacological management and other concomitant treatments precluded control for these potential confounders, which may have influence.

### 4.5. Future Research

Further research is needed to examine whether PPT values and SEL-derived tissue deformation measures change over time and in response to interventions in women with fibromyalgia, using longitudinal and interventional designs. Such studies should incorporate standardized acquisition protocols and formal intra- and inter-rater reliability assessment for SEL to establish measurement reproducibility given the operator-dependent, semi-quantitative nature of the technique before evaluating clinical utility. It would also be useful to incorporate tools that assess psychological variables, physical activity levels, and muscular condition in order to explore their potential correlations with pressure pain threshold and SEL measures in women with FM, and to determine whether these variables explain site-dependent heterogeneity in SEL outcomes without implying direct mechanistic or pathological inferences. In addition, systematically recording potential confounders related to clinical management (e.g., pharmacological treatment and other concomitant therapies) would strengthen interpretability of future findings.

## 5. Conclusions

In this cross-sectional study, women with FM showed lower PPT and site-dependent differences in SEL measures at standardized tender-point sites compared with healthy controls. Given the exploratory design, the operator-dependence, semi-quantitative nature of SEL, and multiple testing across anatomical sites, these findings should be interpreted cautiously and do not establish causal or mechanisms interpretations. Future longitudinal and interventional studies incorporating standardized reliability assessment and predefined primary outcomes are warranted to confirm the observed patterns and clarify their clinical relevance.

Further research should also examine the joint contribution of psychological variables, physical activity, muscle condition, and pain sensitivity to better characterize interactions between central mechanisms and SEL-derived tissue deformation features, without implying direct peripheral pathology in chronic pain.

## Figures and Tables

**Table 1:** Sociodemographic Characteristics.

FM Group *n* = 42; Control Group *n* = 42				
Variable	Group	Mean/Median	SD/IQR	W of Shapiro–Wilk (*p)*
Age **	FM	52.00	12.00	0.96 (0.018)
	Control	52.50	15.00	
Height (m)	FM	1.63	0.04	0.98 (0.472)
	Control	1.63	0.05	
Weight ** (kg)	FM	77.00	34.50	0.96 (0.017)
	Control	65.00	10.00	
Premenopausal	FM	29		
	Control	29		
Postmenopausal	FM	13		
	Control	13		

Abbreviations: SD (standard deviation); IQR (interquartile range); ** (variables that did not follow a normal distribution were represented as Median and IQR); FM (fibromyalgia).

**Table 2:** Descriptive statistics of pressure pain threshold (PPT).

FM Group *n* = 42; Control Group *n* = 42				
Variable	Group	Mean/Median	SD/IQR	W of Shapiro–Wilk (*p*)
Occiput D **	FM	1.35	0.72	0.95 (0.03)
	Control	4.10	1.17	
Occiput ND	FM	1.50	0.80	0.97 (0.17)
	Control	4.22	1.01	
Low cervical D	FM	1.37	0.57	0.97 (0.10)
	Control	3.89	0.87	
Low cervical ND	FM	1.37	0.67	0.97 (0.12)
	Control	3.79	0.81	
Trapezius D **	FM	1.80	0.85	0.90 (<0.00)
	Control	4.10	0.95	
Trapezius ND **	FM	1.50	0.60	0.95 (0.00)
	Control	4.10	1.15	
Supraspinatus D	FM	2.12	1.03	0.98 (0.26)
	Control	6.74	1.81	
Supraspinatus ** ND	FM	1.80	0.77	0.96 (0.02)
	Control	6.85	1.85	
Paraspinous D	FM	2.36	0.95	0.98 (0.22)
	Control	7.39	1.83	
Paraspinous ND	FM	2.59	1.03	0.98 (0.23)
	Control	7.36	1.69	
Gluteus D	FM	2.05	1.32	0.98 (0.54)
	Control	5.94	2.15	
Gluteus ND	FM	1.79	1.08	0.99 (0.94)
	Control	5.72	1.99	
Lateral pectoral D **	FM	1.45	1.00	0.95 (0.00)
	Control	4.10	1.22	
Lateral pectoral ND	FM	1.74	0.84	0.97 (0.15)
	Control	4.40	1.21	
Second rib D **	FM	0.40	1.70	0.71 (<0.00)
	Control	4.00	1.05	
Second rib ND **	FM	0.30	1.00	0.93 (<0.00)
	Control	4.00	1.00	
Lateral epicondyle D **	FM	1.55	1.70	0.93 (<0.00)
	Control	4.45	1.22	
Lateral epicondyle ND **	FM	1.35	1.22	0.95 (0.00)
	Control	4.60	1.47	
Medial epicondyle D **	FM	1.35	1.32	0.96 (0.03)
	Control	4.30	1.07	
Medial epicondyle ND	FM	1.40	1.04	0.98 (0.23)
	Control	4.11	1.25	
Greater trochanter D	FM	1.81	1.25	0.98 (0.71)
	Control	6.02	1.94	
Greater trochanter ND	FM	1.92	1.41	0.99 (0.84)
	Control	6.04	1.98	
Knee D **	FM	1.50	1.65	0.96 (0.02)
	Control	5.35	2.00	
Knee ND **	FM	1.20	1.55	0.96 (0.03)
	Control	5.45	1.77	
Forearm D **	FM	1.05	2.00	0.96 (0.01)
	Control	6.10	1.67	
Forearm ND **	FM	1.45	1.20	0.96 (0.02)
	Control	6.50	1.77	

Abbreviations: D (dominant); ND (non-dominant); SD (standard deviation); IQR (interquartile range); ** (variables that did not follow a normal distribution were represented as Median and IQR).

**Table 3:** Descriptive statistics of strain elastography (SEL).

FM Group *n* = 42; Control Group *n* = 42				
Variable	Group	Mean/Median	SD/IQR	W of Shapiro–Wilk (*p*)
Occiput D	FM	2.40	1.06	0.97 (0.16)
	Control	3.10	0.68	
Occiput ND **	FM	2.20	1.35	0.90 (<0.00)
	Control	2.80	0.63	
Low cervical D **	FM	2.00	1.02	0.93 (<0.00)
	Control	2.50	0.76	
Low cervical ND **	FM	2.15	1.35	0.96 (0.02)
	Control	2.46	0.61	
Trapezius D **	FM	2.00	1.85	0.92 (<0.00)
	Control	3.00	0.60	
Trapezius ND **	FM	2.15	1.72	0.94 (<0.00)
	Control	3.17	0.52	
Supraspinatus D **	FM	2.25	0.95	0.96 (0.01)
	Control	2.93	0.55	
Supraspinatus ND **	FM	2.05	0.90	0.96 (0.01)
	Control	2.68	0.50	
Paraspinous D **	FM	2.75	1.72	0.94 (0.00)
	Control	3.08	0.53	
Paraspinous ND	FM	2.49	1.15	0.97 (0.18)
	Control	2.87	0.51	
Gluteus D	FM	1.70	0.68	0.97 (0.12)
	Control	2.03	0.36	
Gluteus ND **	FM	1.40	0.92	0.91 (<0.00)
	Control	1.98	0.41	
Lateral pectoral D **	FM	1.95	1.02	0.95 (0.00)
	Control	2.03	0.60	
Lateral pectoral ND **	FM	2.20	1.35	0.93 (<0.00)
	Control	2.10	0.32	
Second rib D	FM	2.33	0.85	0.99 (0.80)
	Control	2.73	0.55	
Second rib ND	FM	2.03	0.86	0.98 (0.22)
	Control	2.76	0.76	
Lateral epicondyle D	FM	1.74	1.02	0.97 (0.05)
	Control	2.57	0.62	
Lateral epicondyle ND **	FM	1.30	1.00	0.88 (<0.00)
	Control	2.58	0.53	
Medial epicondyle D **	FM	1.20	1.20	0.93 (<0.00)
	Control	2.46	0.82	
Medial epicondyle ND **	FM	1.40	1.65	0.95 (0.00)
	Control	2.36	0.73	
Greater trochanter D **	FM	1.50	1.22	0.85 (<0.00)
	Control	2.12	0.56	
Greater trochanter ND **	FM	1.40	1.85	0.97 (<0.00)
	Control	2.04	0.48	
Knee D **	FM	1.15	0.80	0.80 (<0.00)
	Control	2.36	0.48	
Knee ND **	FM	1.30	0.85	0.92 (<0.00)
	Control	2.29	0.65	
Forearm D	FM	2.46	1.13	0.98 (0.37)
	Control	2.73	0.41	
Forearm ND **	FM	2.40	2.10	0.96 (0.03)
	Control	2.68	0.37	

Abbreviations: D (dominant); ND (non-dominant); SD (standard deviation); IQR (interquartile range); ** (variables that did not follow a normal distribution were represented as Median and IQR).

**Table 4:** Between-Group Differences in Pressure Pain Threshold (PPT) Variables.

Variable	*p* (Raw)	*p* (FDR-BH)	Mean Difference	SE	95% CI	Effect Sizes (Cohen’s d)
Occiput ND	<0.001 *	<0.001 *	−2.73	0.20	[−3.13, −2.32]	−2.97 ***
Low cervical D	<0.001 *	<0.001 *	−2.52	0.16	[−2.85, −2.19]	−3.43 ***
Low cervical ND	<0.001 *	<0.001 *	−2.42	0.16	[−2.75, −2.09]	−3.30 ***
Supraspinatus D	<0.001 *	<0.001 *	−4.62	0.32	[−5.28, −3.97]	−3.15 ***
Paraspinous D	<0.001 *	<0.001 *	−5.03	0.32	[−5.68, −4.38]	−3.43 ***
Paraspinous ND	<0.001 *	<0.001 *	−4.77	0.31	[−5.39, −4.15]	−3.35 ***
Gluteus D	<0.001 *	<0.001 *	−3.90	0.39	[−4.69, −3.11]	−2.18 ***
Gluteus ND	<0.001 *	<0.001 *	−3.93	0.35	[−4.64, −3.22]	−2.45 ***
Lateral pectoral ND	<0.001 *	<0.001 *	−2.66	0.23	[−3.12, −2.20]	−2.52 ***
Medial epicondyle ND	<0.001 *	<0.001 *	−2.71	0.25	[−3.22, −2.20]	−2.36 ***
Greater trochanter D	<0.001 *	<0.001 *	−4.22	0.36	[−4.94, −3.49]	−2.55 ***
Greater trochanter ND	<0.001 *	<0.001 *	−4.12	0.38	[−4.88, −3.36]	−2.36 ***
Occiput D **	<0.001 *	<0.001 *	−2.60		[−2.90, −2.20]	−3.17 ***
Trapezius D **	<0.001 *	<0.001 *	−2.40		[−2.70, −1.90]	−2.56 ***
Trapezius ND **	<0.001 *	<0.001 *	−2.60		[−2.90, −2.30]	−3.70 ***
Supraspinatus ND **	<0.001 *	<0.001 *	−4.86		[−5.40, −4.30]	−3.77 ***
Lateral pectoral D **	<0.001 *	<0.001 *	−2.90		[−3.30, −2.50]	−3.10 ***
Second rib D **	<0.001 *	<0.001 *	−3.30		[−3.70, −2.60]	−2.56 ***
Second rib ND **	<0.001 *	<0.001 *	−3.40		[−3.80, −2.90]	−3.23 ***
Lateral epicondyle D **	<0.001 *	<0.001 *	−2.80		[−3.40, −2.40]	−2.39 ***
Lateral epicondyle ND **	<0.001 *	<0.001 *	−3.00		[−3.60, −2.50]	−2.33 ***
Medial epicondyle D **	<0.001 *	<0.001 *	−3.00		[−3.50, −2.60]	−2.85 ***
Knee D **	<0.001 *	<0.001 *	−3.90		[−4.50, −3.20]	−2.56 ***
Knee ND **	<0.001 *	<0.001 *	−4.00		[−4.60, −3.30]	−2.63 ***
Forearm D **	<0.001 *	<0.001 *	−4.50		[−5.10, −3.90]	−3.20 ***
Forearm ND **	<0.001 *	<0.001 *	−5.00		[−5.60, −4.40]	−3.56 ***

Abbreviations: D (dominant); ND (non-dominant); * significant level: *p* (raw) < 0.05 and *p* (FDR-BH) < 0.05; SE (Standard error); ** (variables that did not follow a normal distribution were analyzed using the Mann–Whitney U test). Adjusted *p*-values were computed using false discovery rate control (Benjamini–Hochberg; q = 0.05) to reduce Type I error due to multiple comparisons. Cohen’s d was calculated to quantify the magnitude of between-group differences: ***: large effect size (d > 0.5).

**Table 5:** Between-Group Differences in Ultrasound strain elastography (SEL) Variables.

Variable	*p* (Raw)	*p* (FDR-BH)	Mean Difference	SE	95% CI	Effect Sizes (Cohen’s d)
Occiput D	<0.001 *	0.001 *	−0.70	0.19	[−1.09, −0.30]	−0.80 ***
Paraspinous ND	0.05	0.065	−0.38	0.19	[−0.77, 0.00]	−0.43
Gluteus D	0.008 *	0.013 *	−0.32	0.12	[−0.56, −0.09]	−0.58 ***
Second rib D	0.015 *	0.021 *	−0.39	0.15	[−0.73, −0.08]	−0.56 ***
Second rib ND	<0.001 *	0.001 *	−0.73	0.18	[−1.08, −0.37]	−0.88 ***
Lateral epicondyle D	<0.001 *	0.001 *	−0.83	0.18	[−1.21, −0.46]	−1.00 ***
Forearm D	0.148	0.16	−0.27	0.18	[−0.64, 0.09]	−0.32
Occiput ND **	0.014 *	0.021 *	−0.53		[−0.93, −0.13]	−0.56 ***
Low cervical D **	<0.001 *	0.001 *	−0.56		[−0.86, −0.29]	−0.84 ***
Low cervical ND **	0.057	0.068	−0.33		[−0.70, 1.26]	−0.14
Trapezius D **	<0.001 *	0.001 *	−0.91		[−1.33, −0.46]	−0.89 ***
Trapezius ND**	0.001 *	0.001 *	−0.90		[−1.33, −0.43]	−0.85 ***
Supraspinatus D **	<0.001 *	0.001 *	−0.78		[−1.06, −0.46]	−1.11 ***
Supraspinatus ND **	<0.001 *	0.001 *	−0.70		[−0.96, −0.43]	−1.12 ***
Paraspinous D **	0.058	0.068	−0.46		[−0.86, 4.54]	−0.07
Gluteus ND **	<0.001 *	0.001 *	−0.60		[−0.80, −0.32]	−1.06 ***
Lateral pectoral D	0.073	0.082	−0.33		[−0.60, 0.04]	−0.44
Lateral pectoral ND	0.745	0.745	0.07		[−0.26, 0.37]	0.09
Lateral epicondyle ND	<0.001 *	0.001 *	−1.20		[−1.50, −0.93]	−1.80 ***
Medial epicondyle D	<0.001 *	0.001 *	−1.10		[−1.43, −0.76]	−1.40 ***
Medial epicondyle ND	<0.001 *	0.001 *	−0.90		[−1.30, −0.46]	−0.91 ***
Greater trochanter D	0.012 *	0.019 *	−0.53		[−0.80, −0.15]	−0.69 ***
Greater trochanter ND	0.017 *	0.023 *	−0.63		[−0.86, −0.16]	−0.76 ***
Knee D	<0.001 *	0.001 *	−1.06		[−1.26, −0.79]	−1.92 ***
Knee ND	<0.001 *	0.001 *	−0.89		[−1.13, −0.59]	−1.40 ***
Forearm ND	0.325	0.338	−0.26		[−0.83, 0.27]	−0.20

Abbreviations: D (dominant); ND (non-dominant); * significant level: *p* (raw) < 0.05 and *p* (FDR-BH) < 0.05; SE (Standard error); ** (variables that did not follow a normal distribution were analyzed using the Mann–Whitney U test). Adjusted *p*-values were computed using false discovery rate control (Benjamini–Hochberg; q = 0.05) to reduce Type I error due to multiple comparisons. Cohen’s d was calculated to quantify the magnitude of between-group differences; ***: large effect size (d > 0.5).

## Data Availability

Data supporting the findings of this study are available from the corresponding author upon reasonable request. The dataset contains sensitive personal information and is therefore not publicly available due to privacy and ethical restrictions.

## Abbreviations

The following abbreviations are used in this manuscript:

FM	Fibromyalgia
IQR	Interquartile range
ND	No dominant
D	Dominant
SD	Standard deviation
ROIs	Represent distinct regions of interest
STROBE	Statement of Recommendations for Observational Studies
PPT	Pressure pain threshold
SEL	Strain elastography
HPA	Hypothalamic–pituitary–adrenal
CNS	Central nervous system
SSS	Symptom Severity Scale
TPs	Tender points
ACR	American College of Rheumatology
WPI	Widespread Pain Index
MRI	Magnetic resonance imaging

## References

[1] Arnold L.M., Bennett R.M., Crofford L.J., Dean L.E., Clauw D.J., Goldenberg D.L., Fitzcharles M.-A., Paiva E.S., Staud R., Sarzi-Puttini P. (2019). AAPT Diagnostic Criteria for Fibromyalgia. J. Pain.

[2] Bair M.J., Krebs E.E. (2020). Fibromyalgia. Ann. Intern. Med..

[3] Wolfe F., Clauw D.J., Fitzcharles M.A., Goldenberg D.L., Häuser W., Katz R.L., Mease P.J., Russell A.S., Russell I.J., Walitt B. (2016). 2016 Revisions to the 2010/2011 fibromyalgia diagnostic criteria. Semin. Arthritis Rheum..

[4] Sosa-Reina M.D., Núñez-Nagy S., Gallego-Izquierdo T., Pecos-Martín D., Monserrat J., Álvarez-Mon M. (2017). Effectiveness of Exercise Therapy in Fibromyalgia Syndrome: A Systematic Review and Meta-Analysis of Randomized Clinical Trials. BioMed Res. Int..

[5] Navarro-Ledesma S., Carroll J., González-Muñoz A., Pruimboom L., Burton P. (2022). Changes in Circadian Variations in Blood Pressure, Pain Pressure Threshold and the Elasticity of Tissue after a Whole-Body Photobiomodulation Treatment in Patients with Fibromyalgia: A Tripled-Blinded Randomized Clinical Trial. Biomedicines.

[6] Jurado-Priego L.N., Cueto-Ureña C., Ramírez-Expósito M.J., Martínez-Martos J.M. (2024). Fibromyalgia: A Review of the Pathophysiological Mechanisms and Multidisciplinary Treatment Strategies. Biomedicines.

[7] Lorente L.C., Ríos M.C.G., Ledesma S.N., Haro R.M.T., Barragán A.C., Correa-Rodríguez M., Ferrándiz M.E.A. (2019). Functional status and body mass index in postmenopausal women with fibromyalgia: A case–control study. Int. J. Environ. Res. Public Health.

[8] De Formación C. (2009). Revista de la Sociedad Española del Dolor. https://www.elsevier.es/resed.

[9] Alciati A., Nucera V., Masala I.F., Giallanza M., La Corte L., Giorgi V., Sarzi-Puttini P., Atzeni F. (2021). One year in review 2021: Fibromyalgia. Clin. Exp. Rheumatol..

[10] Fitzcharles M.-A., Rampakakis E., Ste-Marie P.A., Sampalis J.S., Shir Y. (2014). The association of socioeconomic status and symptom severity in persons with fibromyalgia. J. Rheumatol..

[11] Queiroz L.P. (2013). Worldwide Epidemiology of Fibromyalgia. Curr. Pain Headache Rep..

[12] Cabo-Meseguer A., Cerdá-Olmedo G., Trillo-Mata J.L. (2017). Fibromyalgia: Prevalence, epidemiologic profiles and economic costs. Med. Clin..

[13] Mas A.J., Carmona L., Valverde M., Ribas B., EPISER Study Group (2008). Prevalence and impact of fibromyalgia on function and quality of life in individuals from the general population: Results from a nationwide study in Spain. Clin. Exp. Rheumatol..

[14] Navarro-Ledesma S., Gonzalez-Muñoz A., Ríos M.C.G., de la Serna D., Pruimboom L. (2022). Circadian Variation of Blood Pressure in Patients with Chronic Musculoskeletal Pain: A Cross-Sectional Study. Int. J. Environ. Res. Public Health.

[15] Wolfe F., Ross K., Anderson J., Russell I.J. (1995). Aspects of fibromyalgia in the general population: Sex, pain threshold, and fibromyalgia symptoms. J. Rheumatol..

[16] Sarzi-Puttini P., Giorgi V., Marotto D., Atzeni F. (2020). Fibromyalgia: An update on clinical characteristics, aetiopathogenesis and treatment. Nat. Rev. Rheumatol..

[17] Di Carlo M., Farah S., Bazzichi L., Atzeni F., Govoni M., Biasi G., Di Franco M., Mozzani F., Gremese E., Dagna L. (2021). Fibromyalgia severity according to age categories: Results of a cross-sectional study from a large national database. Clin. Exp. Rheumatol..

[18] Engel C.C. (2014). Review: In neuropathy, fibromyalgia, or chronic pain, duloxetine reduces pain but increases adverse events. Ann. Intern. Med..

[19] Chinn S., Caldwell W., Gritsenko K. (2016). Fibromyalgia Pathogenesis and Treatment Options Update. Curr. Pain Headache Rep..

[20] García Rodríguez D.F., Abud Mendoza C. (2020). Physiopathology of fibromyalgia. Reumatol. Clin..

[21] Siracusa R., Di Paola R., Cuzzocrea S., Impellizzeri D. (2021). Fibromyalgia: Pathogenesis, mechanisms, diagnosis and treatment options update. Int. J. Mol. Sci..

[22] Navarro-Ledesma S., Aguilar-García M., González-Muñoz A., Pruimboom L., Aguilar-Ferrándiz M.E. (2022). Do Psychological Factors Influence the Elastic Properties of Soft Tissue in Subjects with Fibromyalgia? A Cross-Sectional Observational Study. Biomedicines.

[23] Jahan F., Nanji K., Qidwai W., Qasim R. (2012). Fibromyalgia syndrome: An overview of pathophysiology, diagnosis and management. Oman Med. J..

[24] Dumolard A., Lefaucheur J.-P., Hodaj E., Liateni Z., Payen J.-F., Hodaj H. (2022). Central Sensitization and Small-fiber Neuropathy Are Associated in Patients With Fibromyalgia. Clin. J. Pain.

[25] Flynn D. (2023). Chronic Pain Syndromes: Fibromyalgia. FP Essent.

[26] Coppieters I., Meeus M., Kregel J., Caeyenberghs K., De Pauw R., Goubert D., Cagnie B. (2016). Relations Between Brain Alterations and Clinical Pain Measures in Chronic Musculoskeletal Pain: A Systematic Review. J. Pain.

[27] Malin K., Littlejohn G.O. (2016). Psychological factors mediate key symptoms of fibromyalgia through their influence on stress. Clin. Rheumatol..

[28] Azarfar A., Ahmed A., Bég S. (2021). Prevalence of Anxiety, Depression, Sleep Disturbance, Fibromyalgia, Obesity, and Gastroesophageal Disease in Patients with Rheumatic Diseases. Curr. Rheumatol. Rev..

[29] Gower C., Trevitt J., Cherry B.J., Zettel-Watson L. (2022). Distress as a mediator for pain and activities of daily living in older adults with fibromyalgia. Front. Med..

[30] Akhtar R., Sherratt M.J., Cruickshank J.K., Derby B. (2011). Characterizing the elastic properties of tissues. Mater. Today.

[31] Nguyen Q.T., Jacobsen T.D., Chahine N. (2017). Effects of Inflammation on Multiscale Biomechanical Properties of Cartilaginous Cells and Tissues. ACS Biomater. Sci. Eng..

[32] Schleip R. (2003). Fascial Plasticity—A New Neurobiological Explanation: Part 1. J. Bodyw. Mov. Ther..

[33] Thornton K.G., Robert M. (2020). Prevalence of Pelvic Floor Disorders in the Fibromyalgia Population: A Systematic Review. J. Obstet. Gynaecol. Can..

[34] Kulshreshtha P., Deepak K.K. (2012). Autonomic Nervous System Profile in Fibromyalgia Patients and Its Modulation by Exercise: A Mini Review. Clin. Physiol. Funct. Imaging.

[35] Muro-Culebras A., Cuesta-Vargas A.I. (2013). Sono-myography and sono-myoelastography of the tender points of women with fibromyalgia. Ultrasound Med. Biol..

[36] Sigrist R.M.S., Liau J., Kaffas A.E., Chammas M.C., Willmann J.K. (2017). Ultrasound elastography: Review of techniques and clinical applications. Theranostics.

[37] Navarro-Ledesma S., Aguilar-García M., González-Muñoz A., Casas-Barragán A., Tapia-Haro R.M. (2023). Association between elasticity of tissue and pain pressure threshold in the tender points present in subjects with fibromyalgia: A cross-sectional study. Sci. Rep..

[38] Navarro-Ledesma S., Gonzalez-Muñoz A. (2021). Short-term effects of 448 kilohertz radiofrequency stimulation on supraspinatus tendon elasticity measured by quantitative ultrasound elastography in professional badminton players: A double-blinded randomized clinical trial. Int. J. Hyperth..

[39] Feng Y.N., Li Y.P., Liu C.L., Zhang Z.J. (2018). Assessing the elastic properties of skeletal muscle and tendon using shearwave ultrasound elastography and MyotonPRO. Sci. Rep..

[40] Navarro-Ledesma S., Pruimboom L., Lluch E., Dueñas L., Horno S.M.-D., Gonzalez-Muñoz A. (2022). The Relationship between Daily Physical Activity, Psychological Factors, and Vegetative Symptoms in Women with Fibromyalgia: A Cross-Sectional Observational Study. Int. J. Environ. Res. Public Health.

[41] Borg-Stein J., Stein J. (1996). Trigger points and tender points: One and the same? Does injection treatment help?. Rheum. Dis. Clin. N. Am..

[42] Li L., Stoop R., Clijsen R., Hohenauer E., Fernández-de-las-Peñas C., Huang Q., Barbero M. (2020). Criteria Used for the Diagnosis of Myofascial Trigger Points in Clinical Trials on Physical Therapy Updated Systematic Review. Clin. J. Pain.

[43] Kozinc Ž., Šarabon N. (2020). Shear-wave elastography for assessment of trapezius muscle stiffness: Reliability and association with low-level muscle activity. PLoS ONE.

[44] Valera-Calero J.A., Sánchez-Jorge S., Buffet-García J., Varol U., Gallego-Sendarrubias G.M., Álvarez-González J. (2021). Is Shear-Wave Elastography a Clinical Severity Indicator of Myofascial Pain Syndrome? An Observational Study. J. Clin. Med..

[45] Fernández-De-Las-Peñas C., Dommerholt J. (2018). International consensus on diagnostic criteria and clinical considerations of myofascial trigger points: A delphi study. Pain Med..

[46] Navarro-Ledesma S., Gonzalez-Muñoz A., Carroll J., Burton P. (2022). Short- and long-term effects of whole-body photobiomodulation on pain, functionality, tissue quality, central sensitisation and psychological factors in a population suffering from fibromyalgia: Protocol for a triple-blinded randomised clinical trial. Ther. Adv. Chronic Dis..

[47] Calvo-Lobo C., Diez-Vega I., Martínez-Pascual B., Fernández-Martínez S., de la Cueva-Reguera M., Garrosa-Martín G., Rodríguez-Sanz D. (2017). Tensiomyography, sonoelastography, and mechanosensitivity differences between active, latent, and control low back myofascial trigger points. Medicine.

[48] Littlejohn G., Guymer E. (2020). Key Milestones contributing to the understanding of the mechanisms underlying fibromyalgia. Biomedicines.

[49] Khoo T., Hill C.L., Hoon E., Whittle S. (2022). Patient Perspectives of Disease Activity, Medications and Substance Use in People with Fibromyalgia. Open Access Rheumatol. Res. Rev..

[50] Karlsson B., Burell G., Kristiansson P., Björkegren K., Nyberg F., Svärdsudd K. (2019). Decline of substance P levels after stress management with cognitive behaviour therapy in women with the fibromyalgia syndrome. Scand. J. Pain.

[51] Martínez-Martos J.M., Correa-Rodríguez M., Rus A., Molina F., Ramírez-Expósito M.J., Aguilar-Ferrandiz M.E. (2019). Altered Serum Oxytocinase and Enkephalin-Degrading Aminopeptidase Activities in Patients With Fibromyalgia. Biol. Res. Nurs..

[52] Rus A., Molina F., Del Moral M.L., Ramírez-Expósito M.J., Martínez-Martos J.M. (2018). Catecholamine and Indolamine Pathway: A Case–Control Study in Fibromyalgia. Biol. Res. Nurs..

[53] Wilson J.M., Meints S.M., Edwards R.R., Yamin J.B., Moore D.J. (2024). The role of sleep disturbance in reduced accuracy on a divided attention task among patients with fibromyalgia. PAIN Rep..

[54] Deutsch G., Deshpande H., Lai H.H., Kutch J.J., Ness T.J. (2021). Cerebral Perfusion and Sensory Testing Results Differ in Interstitial Cystitis/Bladder Pain Syndrome Patients with and without Fibromyalgia: A Site-Specific MAPP Network Study. J. Pain Res..

[55] Zheng C., Zhou T. (2022). Effect of Acupuncture on Pain, Fatigue, Sleep, Physical Function, Stiffness, Well-Being, and Safety in Fibromyalgia: A Systematic Review and Meta-Analysis. J. Pain Res..

[56] Benlidayi I.C. (2019). Role of inflammation in the pathogenesis and treatment of fibromyalgia. Rheumatol. Int..

[57] Littlejohn G., Guymer E. (2018). Neurogenic inflammation in fibromyalgia. Semin. Immunopathol..

[58] Karayol K.C., Karayol S.S. (2021). A comparison of visual analog scale and shear-wave ultrasound elastography data in fibromyalgia patients and the normal population. J. Phys. Ther. Sci..

[59] Eby S.F., Song P., Chen S., Chen Q., Greenleaf J.F., An K.-N. (2013). Validation of shear wave elastography in skeletal muscle. J. Biomech..

[60] Murillo C., Falla D., Rushton A., Sanderson A., Heneghan N.R. (2019). Shear wave elastography investigation of multifidus stiffness in individuals with low back pain. J. Electromyogr. Kinesiol..

[61] Turo D., Otto P., Hossain M., Gebreab T., Armstrong K., Rosenberger W.F., Shao H., Shah J.P., Gerber L.H., Sikdar S. (2015). Novel Use of Ultrasound Elastography to Quantify Muscle Tissue Changes After Dry Needling of Myofascial Trigger Points in Patients With Chronic Myofascial Pain. J. Ultrasound Med..

[62] Sancar M., Keniş-Coşkun Ö., Gündüz O.H., Kumbhare D.M. (2021). Quantitative Ultrasound Texture Feature Changes With Conservative Treatment of the Trapezius Muscle in Female Patients With Myofascial Pain Syndrome. Am. J. Phys. Med. Rehabil..

